# Whole-Genome Sequence of Corynebacterium xerosis Strain GS1, Isolated from Yak in Gansu Province, China

**DOI:** 10.1128/MRA.00556-19

**Published:** 2019-09-12

**Authors:** Fengqin Wen, Xiaoyong Xing, Shijun Bao, Yonghao Hu, Bao-cheng Hao

**Affiliations:** a Veterinary Medicine College, Gansu Agricultural University, Gansu Province, China; b Lanzhou Institute of Animal and Veterinary Pharmaceutics Science, Chinese Academy of Agriculture Science, Gansu Province, China; Loyola University Chicago

## Abstract

We report here the isolation, sequencing of the complete closed genome, and annotation of Corynebacterium xerosis strain GS1. This strain was isolated from the liver lesion of a yak in Gansu Province, China. The genome consists of one chromosome with 2,738,835 bp and comprises 2,304 protein-coding genes.

## ANNOUNCEMENT

The genus *Corynebacterium*, which currently has more than 110 validated species, is highly diversified. It includes species that are of medical, veterinary, and biotechnological relevance (1).

Corynebacterium xerosis is a commensal organism present in the skin and mucous membranes of humans (2). The species *C. xerosis* is also a frequently reported human pathogen, with isolates being identified in cases that include ear infections, brain abscesses, osteomyelitis, and maternal ventriculoperitoneal shunt infections (3, 4). It has also been isolated in pure culture from normally sterile organs of animal clinical specimens. *C. xerosis* was isolated from a pig’s joint suffering from a subcutaneous abscess and from a goat liver suspected to have paratuberculosis (5). The first case for *C. xerosis* producing a clinical cutaneous abscess in sheep was reported in Mexico in 2016 (6).

*C. xerosis* is phylogenetically closely related to Corynebacterium freneyi, Corynebacterium amycolatum, and Corynebacterium hansenii (7–9). These species have similar colony morphologies and biochemical characteristics. The difficulty of its correct phenotype-based identification in routine clinical microbiology laboratories may imply that the clinical significance of *C. xerosis* in veterinary medicine could be higher than currently considered (10).

The results of this study provide further information about biological and genetic characteristics which not only contribute to better identification between these closely related species but also encourage further exploration of its pathogenesis as a zoonotic pathogen.

**FIG 1:**
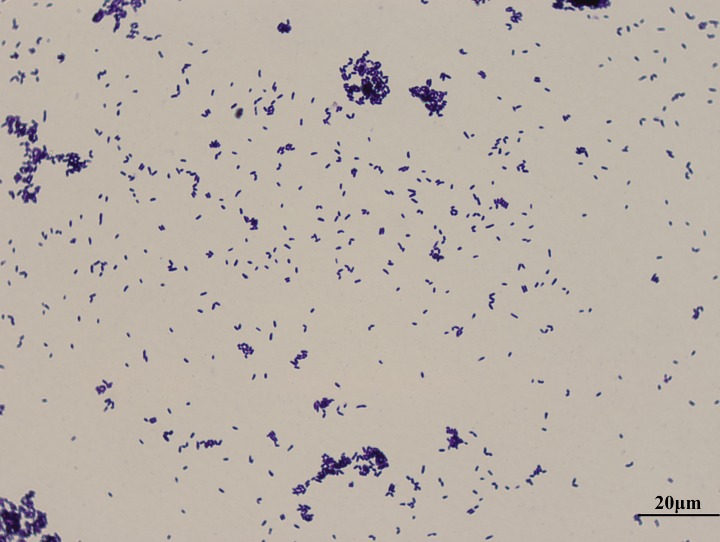
Gram-stained smear preparation of *C. xerosis* (×1,000 magnification).

In this study, the *C. xerosis* strain GS1 was isolated in 2015 from a liver lesion with caseous nodules from a yak. First, the liver tissue was surface disinfected and inoculated on nutrient agar with 5% sheep blood for 24 h at 37°C. Then, the morphologically different colonies were recultured and identified according to 16S rRNA sequencing (11). The *C. xerosis* colony was yellow-gray and dry with an irregular edge. Gram-positive, rod-shaped bacteria were observed under the microscope after Gram staining of the smear (Fig. 1).

A purified single colony of *C. xerosis* strain GS1 was inoculated into Luria broth (LB) and cultured at 37°C for 24 h prior to DNA extraction. Many insoluble particles can be seen in LB.

DNA extraction was performed using the Wizard genomic DNA purification kit (Promega), according to the manufacturer’s instructions. The library was constructed with the SMRTbell template prep kit 1.0 (Pacific Biosciences). Single-molecule real-time (SMRT) sequencing was performed on the PacBio Sequel platform at Shanghai OE Biotech Corporation (Shanghai, China).

The PacBio sequencing yielded a total of 155,567 reads, with a mean read length of 4,003 nucleotides. The *N*_50_ value is 6,132 bp. These reads were subsequently used for *de novo* assembly with FALCON v0.3.0 (12). Default settings were used for each program.

The consensus assembly generated one contig of 2,738,835 bp (220-fold coverage). The contig was circularized using Circlator v1.1.2 (13). The average chromosome G+C content was 69.29%. No prophage was found in this strain when PhiSpy v2.3 (14) was used.

Gene prediction and functional annotation were processed using the NCBI Prokaryotic Genome Annotation Pipeline (15, 16) and GeneMarkS+ annotation system (17). A total of 2,423 genes were predicted, including 2,304 protein-coding genes, 65 RNA genes (12 rRNAs, 50 tRNAs, and 3 noncoding RNAs [ncRNAs]), and 54 pseudogenes.

### Data availability.

The sequence and annotation of Corynebacterium xerosis strain GS1 have been deposited in GenBank under accession number CP032788. The raw read accession number is SRX6372980. The version described in this paper is the first version. The BioSample number is SAMN10065701, and the BioProject number is PRJNA491202.
